# Diagnosis of Schizophrenia Using Feature Extraction from EEG Signals Based on Markov Transition Fields and Deep Learning

**DOI:** 10.3390/biomimetics10070449

**Published:** 2025-07-07

**Authors:** Alka Jalan, Deepti Mishra, Marisha, Manjari Gupta

**Affiliations:** 1 Department of Computer Science, Institute of Science, Banaras Hindu University, Varanasi 221005, India; jalanalka5@bhu.ac.in (A.J.); marisha@bhu.ac.in (M.); manjari@bhu.ac.in (M.G.); 2 Department of Computer Science, Faculty of Information Technology and Electrical Engineering, Norwegian University of Science and Technology, 2815 Gjøvik, Norway

**Keywords:** schizophrenia, Markov Transition Field, VGG-16, electroencephalogram, deep learning, explainability

## Abstract

Diagnosing schizophrenia using Electroencephalograph (EEG) signals is a challenging task due to the subtle and overlapping differences between patients and healthy individuals. To overcome this difficulty, deep learning has shown strong potential, especially given its success in image recognition tasks. In many studies, one-dimensional EEG signals are transformed into two-dimensional representations to allow for image-based analysis. In this work, we have used the Markov Transition Field for converting EEG signals into two-dimensional images, capturing both the temporal patterns and statistical dynamics of the data. EEG signals are continuous time-series recordings from the brain, where the current state is often influenced by the immediately preceding state. This characteristic makes MTF particularly suitable for representing such data. After the transformation, a pre-trained VGG-16 model is employed to extract meaningful features from the images. The extracted features are then passed through two separate classification pipelines. The first uses a traditional machine learning model, Support Vector Machine, while the second follows a deep learning approach involving an autoencoder for feature selection and a neural network for final classification. The experiments were conducted using EEG data from the open-access Schizophrenia EEG database provided by MV Lomonosov Moscow State University. The proposed method achieved a highest classification accuracy of 98.51 percent and a recall of 100 percent across all folds using the deep learning pipeline. The Support Vector Machine pipeline also showed strong performance with a best accuracy of 96.28 percent and a recall of 97.89 percent. The proposed deep learning model represents a biomimetic approach to pattern recognition and decision-making.

## 1. Introduction

Schizophrenia (SCZ) is a chronic and severe mental disorder that impacts around 20 million individuals globally [1]. It is characterized by long-term disturbances in brain function, presenting symptoms such as persistent false beliefs (delusions), sensory perceptions without external stimuli (hallucinations), and disorganized emotions, perceptions, or speech [2]. In comparison to the general population, individuals with schizophrenia face a higher mortality rate, often due to preventable physical health conditions [3]. It is typically diagnosed through clinical evaluation based on standardized criteria obtained during one-on-one interviews. However, the reliability of such diagnoses can vary due to differences in the qualifications, experience, and time constraints of the mental health professionals conducting the assessments [4]. Therefore, there is a need for EEG-based biomarkers that can potentially identify early or prodromal stages of schizophrenia, even before full-blown symptoms appear. Moreover, EEG is relatively affordable, portable, and non-invasive, making it suitable for large-scale screenings or resource-limited settings as compared to neuroimaging techniques like fMRI (functional Magnetic Resonance Imaging), which are costly [5].

EEG signals can be analyzed using AI models to identify patterns not visible to the human eye, improving diagnostic precision and enabling personalized medicine. Although artificial intelligence (AI) has seen widespread adoption in physical healthcare, its application in the field of mental health remains considerably limited [6]. Given the increasing demand for objective, efficient, and reproducible diagnostic tools in mental health, the integration of artificial intelligence (AI) into schizophrenia detection has shown promising advancements. Prior studies have explored various deep learning and machine learning architectures, such as CNNs with time-series image transformations, graph-based models like GCN-LSTM [7], and hybrid approaches involving wavelet transforms [8] and statistical features [9]. These methods have demonstrated the potential of EEG-based AI models in capturing subtle neural patterns associated with schizophrenia. Building on these foundations, this study aims to explore a robust and interpretable classification framework by utilizing Markov Transition Field (MTF) representations of EEG signals. These 2D transformations are further processed using VGG16 for high-level feature extraction. The extracted features are subsequently classified using Support Vector Machines (SVM) and an Autoencoder-based Neural Network. Through this integration, the study seeks to contribute to the development of accurate and scalable AI-driven diagnostic tools in psychiatric research. Finally, SHAP stands for SHapley Additive exPlanations, a method in Explainable AI (XAI) that uses game theory to explain the output of AI models, which has been used to incorporate explainability in our proposed method. In this study, we utilized a biomimetic computational approach to address challenges in the diagnosis of psychiatric disorders, which often involve subtle and complex symptom patterns. By employing a deep learning framework—specifically, an autoencoder paired with a Fully Connected Neural Network—we emulate the brain’s ability to compress, abstract, and decode patterns in data.

The main contributions of the work are as follows:

- We are the first to integrate Markov Transition Fields (MTF) with deep feature extraction (VGG-16) on EEG data for schizophrenia detection.
- We implemented a traditional Support Vector Machine (SVM) and a Neural Network (NN) integrated with an autoencoder for dimensionality reduction, ensuring both accuracy and computational efficiency.
- We have performed a comprehensive evaluation of the model performance, including accuracy, precision, recall, specificity, F1-score, and AUC to validate the reliability of the proposed architecture.
- We have demonstrated the model’s explainability and generalizability by plotting a SHAP (SHapley Additive exPlanations) plot for our autoencoder with a neural network model, positioning it as a scalable tool for aiding clinical decision-making in mental health diagnostics.

The paper is organized in the following manner: Section 2 shows related work, Section 3 describes detailed methodology, Section 4 demonstrates results and analysis, Section 5 shows the discussion, and Section 6 finally talks about conclusion and future works.

## 2. Related Work

Previous research has investigated the use of time-frequency transformations of EEG data for classifying schizophrenia. A deep learning model utilized spectrogram images of EEG signals for the detection of schizophrenia [10]. This method involved converting raw EEG signals into two-dimensional images using the Short-Time Fourier Transform (STFT), allowing the extraction of informative time–frequency features. Unlike traditional techniques that depend on handcrafted features, this approach utilizes the detailed patterns present in 2D representations. The authors used two datasets. The first dataset used in the study comprises EEG recordings from 39 healthy control subjects and 45 children diagnosed with a similar type of schizophrenia, with diagnoses confirmed by experts from the Mental Health Research Center (MHRC). The second dataset includes EEG recordings from 14 healthy individuals and 14 patients with schizophrenia. This data was collected by the Institute of Psychiatry and Neurology in Warsaw, Poland, and includes an equal number of male and female participants, with average ages of 27.3 ± 3.3 years for males and 28.3 ± 4.1 years for females. Deep features were extracted from these STFT images using a pre-trained VGG-16 convolutional neural network, achieving classification accuracies of 95% and 97% across different age groups of schizophrenia patients and healthy individuals. This work was among the earliest to combine 2D time–frequency feature extraction with deep learning for schizophrenia detection, highlighting the potential of image-based methods in extracting complex EEG characteristics.

The authors [11] explored a novel approach for diagnosing schizophrenia by converting EEG time series data into image-based representations. Their study focused on the N100 EEG component, previously shown to differ between individuals with schizophrenia and healthy controls. EEG recordings from 81 participants—comprising 49 schizophrenia patients and 32 healthy individuals—were transformed into two-dimensional images using Recurrence Plot (RP) and Gramian Angular Field (GAF) techniques. These image-based representations were then fed into convolutional neural networks (CNNs) inspired by the VGGNet architecture. The EEG dataset was provided by the National Institute of Mental Health (NIMH; R01MH058262). Among the tested methods, the model trained on EEG graph images achieved an accuracy of 75.3%, while RP and GAF-based models demonstrated superior performance with classification accuracies of 90% and 93.2%, respectively, underscoring the effectiveness of time series image conversion in capturing complex EEG patterns for schizophrenia detection.

In [12], the researchers employed phase space dynamics (PSD) derived from EEG signals to distinguish between individuals diagnosed with schizophrenia and healthy controls. Two-dimensional PSD representations were made in Cartesian coordinates, from which fifteen graphical features were extracted to reflect the inherent chaotic properties of EEG activity. Feature relevance and optimal electrode selection were made using the forward selection algorithm (FSA). The dataset used in this study comprised EEG data from 14 schizophrenia patients and 14 healthy subjects, with an equal distribution of male and female participants. This data was sourced from the Institute of Psychiatry and Neurology in Warsaw, Poland. The authors implemented eight different classification models for their effectiveness in detecting schizophrenia. Among these, the K-Nearest Neighbor (KNN) classifier using City-block distance and the Generalized Regression Neural Network (GRNN) yielded the best results. The KNN model, evaluated using 10-fold cross-validation, gave an average accuracy of 94.80%, a sensitivity of 94.30%, and a specificity of 95.20%.

In another research article [9], EEG signals were decomposed into multiple sub-band components using a Fourier-based method, specifically employing the Fast Fourier Transform (FFT) for real-time implementation. From these sub-bands, statistical features were extracted to characterize signal behavior. Furthermore, a Look Ahead Pattern (LAP) feature was introduced to effectively capture localized variations within the EEG signals. This dual approach facilitated a more comprehensive analysis of the underlying data. Feature selection was performed using the Kruskal–Wallis test to identify the most discriminative attributes. The dataset is the same as in [8]. Several machine learning classifiers were examined, and the proposed methodology, when paired with a Boosted Trees classifier, achieved a high classification accuracy of 98.62% for identifying schizophrenia.

In the study [13], brain rhythm data—known for their effectiveness in analyzing diverse brain activities—were transformed into two-dimensional images for input into a deep learning framework. The Markov Transition Field (MTF) technique was used to generate these images, effectively preserving the temporal and statistical dynamics inherent in EEG signals, which are essential for distinguishing between different seizure types. A Convolutional Neural Network (CNN) was then employed for classification. The researchers also explored how image resolution and the choice of specific brain rhythms influenced performance. For experimental purposes, EEG recordings corresponding to six seizure types were sourced from the Temple University Hospital EEG dataset (TUH v1.5.2). The proposed method achieved a peak classification accuracy of 91.1% and a weighted F1-score of 91.0%. Further findings demonstrated that enhancing image resolution led to better classification outcomes.

Recent advancements have demonstrated the effectiveness of graph-based techniques in classification problems, particularly for identifying schizophrenia. A prominent study [7] introduced a hybrid deep learning model that integrates Graph Convolutional Networks (GCN) with Long Short-Term Memory (LSTM) networks (GCN-LSTM) to distinguish between schizophrenia patients and healthy individuals. This model was applied to EEG data collected by the Institute of Psychiatry and Neurology in Warsaw, Poland. The EEG signals were pre-processed and segmented into intervals of 5 and 8 s. From each segment, 14 features were extracted—half from the time domain and half from the frequency domain. EEG electrodes were treated as graph nodes, and signal interactions were modeled as edges to form input graphs for the GCN-LSTM model. A 5-fold cross-validation strategy and various seed values were used to ensure model robustness and avoid overfitting. The model delivered notable results, achieving an average accuracy of 99.25 ± 0.24% on 8 s segments. Additional metrics included a precision of 99.28 ± 0.22%, F1-score of 99.24 ± 0.24%, sensitivity of 99.67 ± 0.28%, specificity of 98.73 ± 0.64%, and an AUC of 99.20 ± 0.27%. Statistical analysis using *t*-tests and ANOVA confirmed that features like zero-crossing rate, Hjorth mobility, peak frequency, and gamma band power significantly contributed to classification. This study highlights the potential of combining graph-structured representations with deep learning to enhance EEG-based psychiatric diagnosis.

## 3. Methodology

In this section, the complete methodology adopted for schizophrenia detection using EEG signals has been described step by step. The detailed process of transforming EEG time-series data into two-dimensional images using the Markov Transition Field (MTF) technique has been explained. This is followed by deep feature extraction using the VGG-16 model, dimensionality reduction via an autoencoder, and final classification using a neural network. The performance of our model with a Support Vector Machine (SVM) classifier is also evaluated.

### 3.1. Dataset

**Figure 1:**
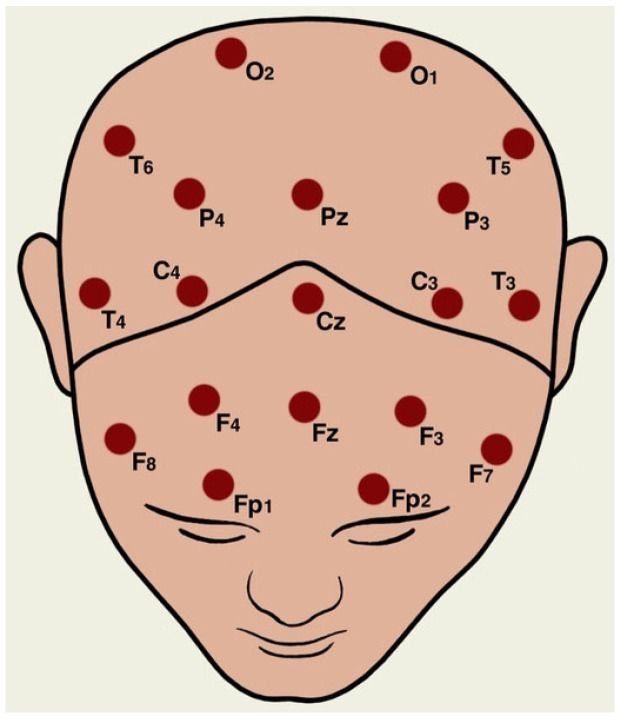
10–20 placement system.

This study uses EEG data from the open-access Schizophrenia EEG database provided by MV Lomonosov Moscow State University [12]. The dataset contains time series values stored in ‘.eea’ format (standard ASCII), recorded using the 10–20 international electrode placement system [13]. The recordings include two groups: 45 adolescents with schizophrenia (aged 10–14) and 39 healthy controls (aged 11–13). EEG signals were collected while participants were resting with their eyes closed. Each recording lasted 1 min with a sampling frequency of 128 Hz, generating EEG segments of 7680 data points after artifact removal. Each value in the column represents the EEG amplitude (µV) recorded at a specific sample point. EEG signals were captured from 16 electrodes, which are in order (O1, O2, P3, P4, Pz, T5, T6, C3, C4, Cz, T3, T4, F3, F4, F7, and F8) using a linked earlobe reference. The impedance of all electrodes was kept below 10 kΩ throughout the recording process. To ensure clean signals, eye blink and movement artifacts were manually removed by two experts, resulting in an artifact-free dataset. Figure 1 below displays the topographical locations of the channel numbers [12].

### 3.2. Preprocessing

Before undergoing the feature extraction phase, the raw EEG data underwent preprocessing. Each EEG recording was provided in single-column ASCII format containing numerical time-series values. The data were reshaped into 16 channels, with each channel containing 7680 samples, corresponding to the 1 min recording duration at a sampling rate of 128 Hz. That means the signals are segmented into epochs, with each epoch containing 7680 points per channel. The dataset was structured into two folders, ‘norm’ for storing data of healthy controls and ‘sch’ for storing data of schizophrenic patients, each containing multiple EEG files. The data-loading process involved iterating through all EEG files and storing them in a structured dictionary format.

### 3.3. Markov Transition Field Transformation

**Figure 2:**
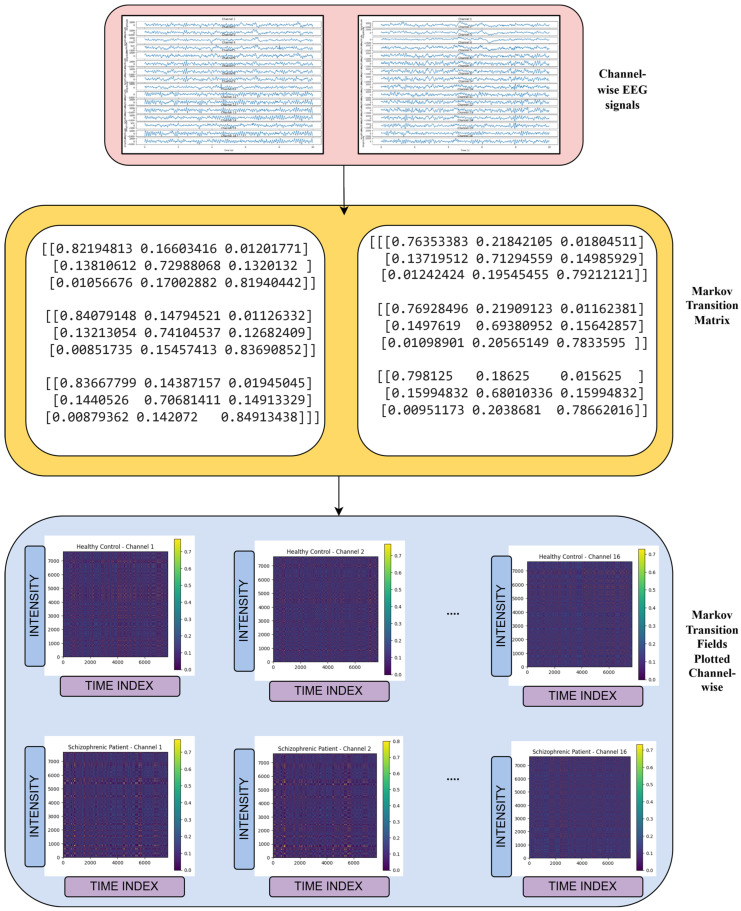
Markov Transition Field image generation.

The Markov Transition Field (MTF) transforms a 1D time series into a 2D representation by capturing the transition probabilities between discretized values over time [14]. During the 2D encoding process, it effectively retains the temporal patterns and statistical dynamics of the time series. EEG signals are continuous time-series data captured from the brain. EEG signals also show patterns where the current state often depends on the immediate previous state due to brain wave continuity and dynamics. In many EEG studies, it is reasonable to approximate EEG dynamics as having Markov-like behavior so that the signal evolution over time can be modeled using transition probabilities between different states in the form of quantized amplitudes [15]. As shown in Figure 2, the transformation process started with reshaping each EEG signal $S\in R^{7680\;}$ into (1,7680) format to comply with MTF transformation requirements. The MTF representation was generated by

(1) $$M_{\left\{i,j\right\}}=P\left(s_{\left\{t\right\}}\to s_{\left\{t+1\right\}}\right|q\left(i\right),q(j))$$

**Figure 3:**
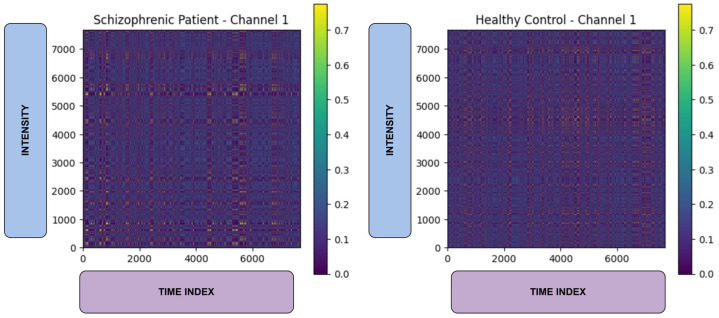
MTF images of schizophrenic (**left**) and healthy control (**right**).

Here, the $P(s_{\left\{t\right\}}\to s_{\left\{t+1\right\}})$ denotes the transition probability between quantized signal values, and *q(i)* and *q(j)* represent the quantile bins of the signal values. The Quantile-based binning with *n* = 8 bins has been used to discretize the signal values, thereby ensuring even distribution across bins. The resulting MTF images were stored as 2D matrices and visualized using the viridis colormap, which is also 2D, to highlight the intensity of transitions. Each channel-level image was saved as a separate PNG file, which consists of three channels, ‘RGB’. The Markov Transition Field (MTF) provides a visual representation of EEG signal dynamics by encoding the transition probabilities between various signal states over time. The MTF can be converted into 2D images, making it suitable for use as input in deep learning models [14]. In individuals with schizophrenia, disruptions in neural activity result in irregular and unstable transitions between these states. The MTF effectively captures these changes, presenting them as distinct patterns that differ significantly from those observed in healthy individuals. EEG signals from patients with schizophrenia often show reduced inter-regional synchrony and chaotic temporal behavior, both of which are reflected in the MTF image. These unique characteristics make the MTF a valuable tool for distinguishing schizophrenia-related EEG patterns from normal brain activity. The total number of MTF images generated is 1344, comprising 624 from ‘norm’ and 720 from ‘sch’ class. Figure 3 represents the MTF images of schizophrenic (**left**) and healthy control (**right**).

### 3.4. Feature Extraction Using Pre-Trained VGG-16 Network

**Figure 4:**
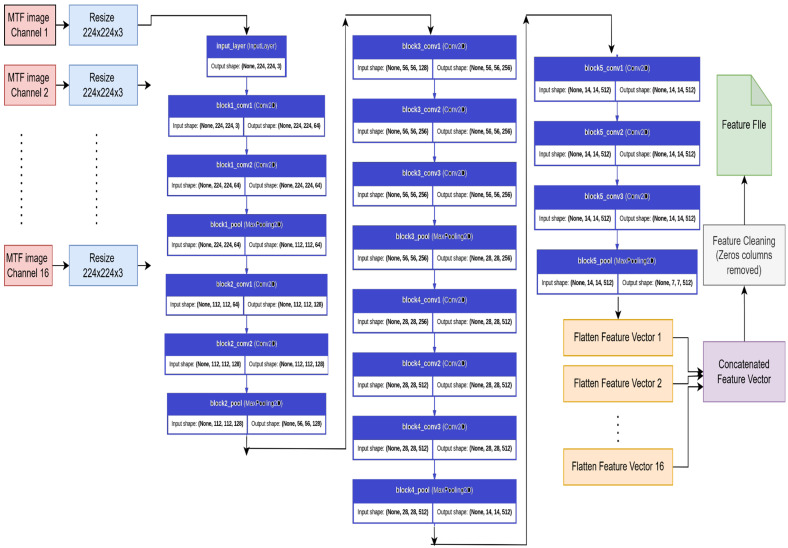
Feature extraction from MTF images using VGG-16.3.5. Experimental design.

To capture higher-level spatial characteristics from EEG signals, the one-dimensional time-series data were first transformed into two-dimensional Markov Transition Field (MTF) images. These images represent the temporal transition patterns of EEG signals in a format well-suited for deep convolutional neural networks. To utilize the expressive power of deep learning, a pre-trained VGG-16 model, as shown in Figure 4, was used as a feature extractor. VGG-16, introduced by Simonyan and Zisserman at the University of Oxford’s Visual Geometry Group in 2014 [16], is a deep convolutional neural network with 13 convolutional layers and 3 fully connected layers. It uses small 3 × 3 kernels and ReLU activations. In this study, the convolutional layers of the VGG-16 model (excluding the final classification layers) were used to extract layered features from the MTF images. The model was pre-trained on the ImageNet dataset, allowing for effective feature extraction through transfer learning, without the need for additional training on the EEG data. Pretrained VGG16 enables effective model building on small datasets by transferring general visual knowledge learned from large-scale data, reducing the need for extensive training, and minimizing the risk of overfitting.

Each MTF image with the size 500 × 500 × 3, corresponding to one of the 16 EEG channels, was resized using a python code to 224 × 224 × 3 to meet the input size requirements of the VGG-16 network. These images were passed through the convolutional layers, and the output from the final layer was flattened to form deep feature vectors. The vectors from all 16 channels were then combined to generate a single feature vector for each subject. In order to maintain the relevance and compactness of the feature set, a cleaning step was applied post-extraction. The columns in the feature matrix that contained zero values for all subjects were removed. This helped eliminate non-informative features and reduced the possibility of overfitting during the classification phase.

The features extracted using the VGG-16 model were saved in a CSV file and later imported for further analysis. Before proceeding with model training, any missing values were addressed by replacing them with the corresponding column means. Class labels were converted into binary format, assigning 0 to the “norm” group and 1 to the “sch” group. To standardize the input data, z-score normalization was applied using StandardScaler, which ensured a mean of zero and a standard deviation of one for every feature.

#### 3.4.1. Model_1: Support Vector Machine (SVM) Classification Using VGG-16 Features

In order to ensure a reliable evaluation of model performance, five-fold cross-validation was implemented. The dataset was randomly divided into five equal parts while maintaining the original class distribution. In each iteration, the Support Vector Machine (SVM) model was trained on 80% of the data and tested on the remaining 20%. The SVM was set up with a radial basis function (RBF) kernel, using a regularization parameter C = 1.0 and γ = “scale”. Additionally, probabilistic predictions were enabled to support the calculation of the AUC-ROC metric.

#### 3.4.2. Model_2: Deep Learning Based Classification Using VGG-16 Features

The experimental framework consisted of two key stages:

- Autoencoder for Feature Compression: A symmetrical autoencoder was designed with gaussian noise injection [17] at the input to enhance generalizability. It is random noise sampled from a gaussian distribution with a mean and a standard deviation. It simulates real-world EEG-variability, forcing the autoencoder to learn noise-invariant features. The encoder comprised two hidden layers with 512 and 256 neurons, respectively, activated using ELU and regularized using L2 weight decay and dropout layers. The encoded latent representation was size 512. The decoder mirrored the encoder architecture in reverse order to reconstruct the input features. The model was trained to minimize mean squared error (MSE) loss. The hyperparameters that are used in the model are batch_size = 64, learning_rate = 0.001, encoding_dim = 512, epochs = 30, and droupout = 0.4.
- Classification with Fully Connected Neural Network: The encoded features were then fed into a Fully Connected Neural Network classifier comprising two hidden layers (128 and 64 neurons) with ReLU activation, batch normalization, L2 regularization, and dropout. The output layer consisted of a single sigmoid unit for binary classification. The model was compiled using the Adam optimizer with a binary cross-entropy loss function. The hyperparameters that are used in the model are batch_size = 64, learning_rate = 0.001, epochs = 30, and droupout = 0.5.

**Figure 5:**
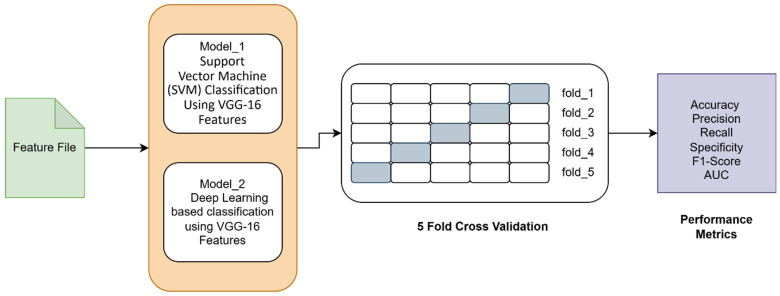
Experimental flow diagram.

The experimental flow diagram is shown in Figure 5.

### 3.5. Hardware and Software

The MTF images were generated using an HP workstation equipped with a 64-bit Intel^®^ Xeon^®^ W7-2475X processor running at 2.59 GHz, 256 GB of installed RAM, and operating with Ubuntu 24.10 for Workstations. The rest of the processing was carried out using Google Colab [18]. The Python libraries like pyts [19], Numpy, Pandas, Matplotlib [20], and Seaborn 0.11.1 were used for data processing and visualization; Scikit-learn for model evaluation and preprocessing; TensorFlow/Keras for deep learning models.

### 3.6. Evaluation Metrics

The effectiveness of the proposed approach was evaluated using standard performance metrics, including the confusion matrix, accuracy, precision, recall, specificity, F1-score, and the area under the curve (AUC).

Confusion matrix: The confusion matrix, shown in Table 1, is used to visualize classification outcomes by presenting true positives (TP), true negatives (TN), false positives (FP), and false negatives (FN).

Accuracy: This metric indicates the overall performance of the model by calculating the ratio of correctly classified instances to the total number of instances.

(2) $$\mathrm{Accuracy}=\left(\mathrm{TP}+\mathrm{TN}\right)/\left(\mathrm{TP}+\mathrm{TN}+\mathrm{FP}+\mathrm{FN}\right)$$

Precision: This metric evaluates the proportion of true positive predictions out of all positive predictions made by the model, reflecting the ability of the model to minimize false positives.

(3) Precision=TP/(TP+FP)

Recall (Sensitivity): This metric measures the proportion of true positives accurately identified by the model, indicating its ability to detect positive cases.

(4) Recall=TP/(TP+FN)

Specificity: This metric calculates the proportion of true negatives correctly identified by the model, reflecting its ability to detect negative cases.

(5) Specificity=TN/(TN+FP)

F1-Score: It combines both precision and recall by taking the harmonic mean of the two. A better F1 score implies improving both precision and recall simultaneously.

(6) $$F_{1}\;\mathrm{Score}=2\times \frac{\mathrm{Precision}\times \mathrm{Recall}}{\mathrm{Precision}+\mathrm{Recall}}$$

Area Under the Receiver Operating Characteristic Curve (AUC-ROC): This assesses the ability of the model to differentiate between classes at various threshold settings. A higher AUC value signifies better discriminative performance.

## 4. Results and Analysis

**Figure 6:**
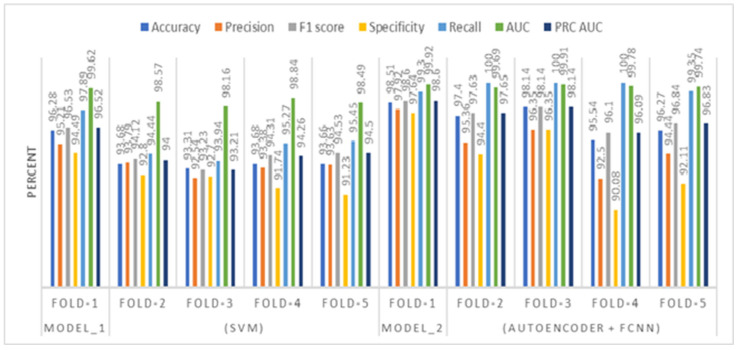
Performance metrics illustration of Model_1 and Model_2.

**Figure 7:**
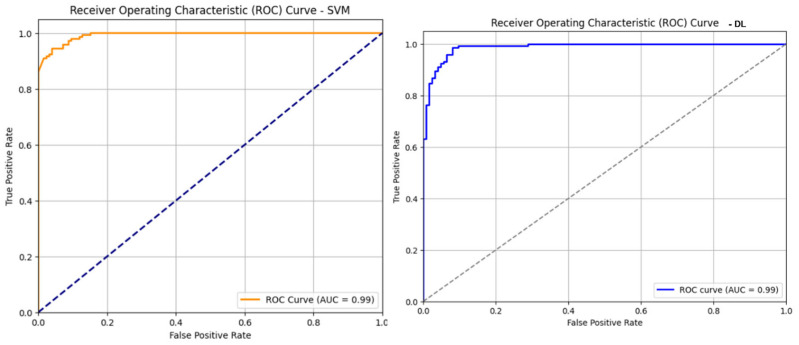
AUC-ROC curve of fold_1 of Model_1 (**left**) and Model_2 (**right**).

**Figure 8:**
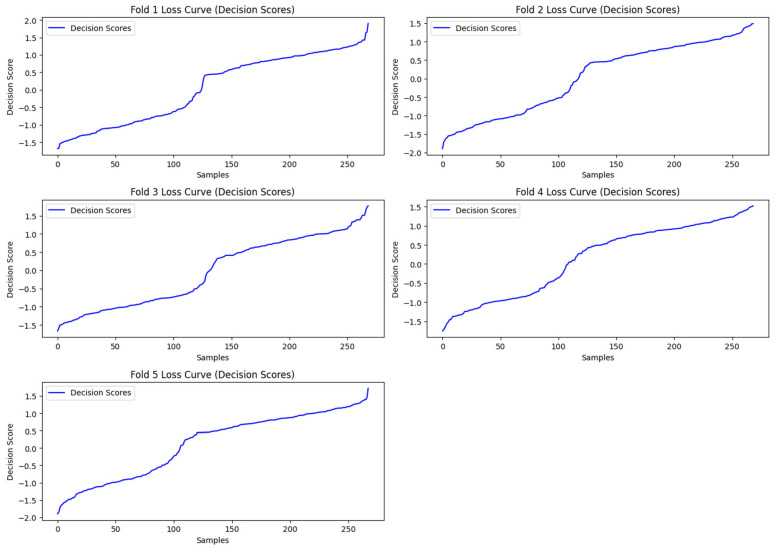
The Loss Function plot varying across five folds of Model_1.

**Figure 9:**
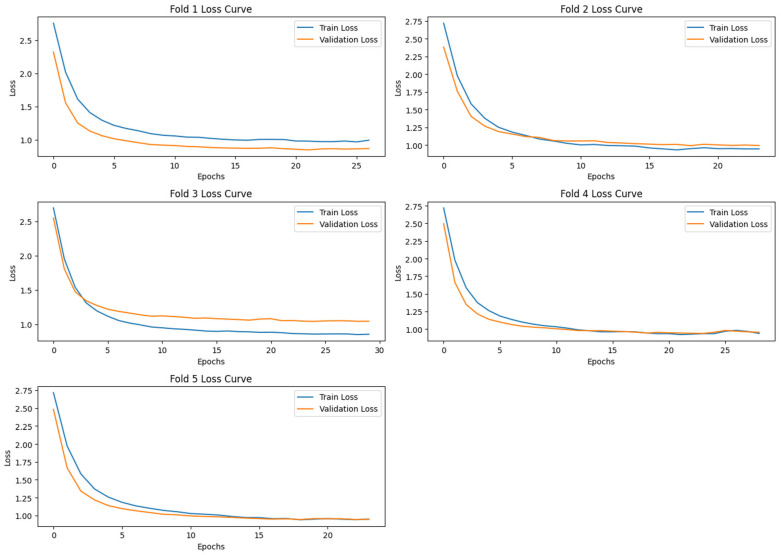
The Loss Function plot varying across five folds of Model_2.

**Figure 10:**
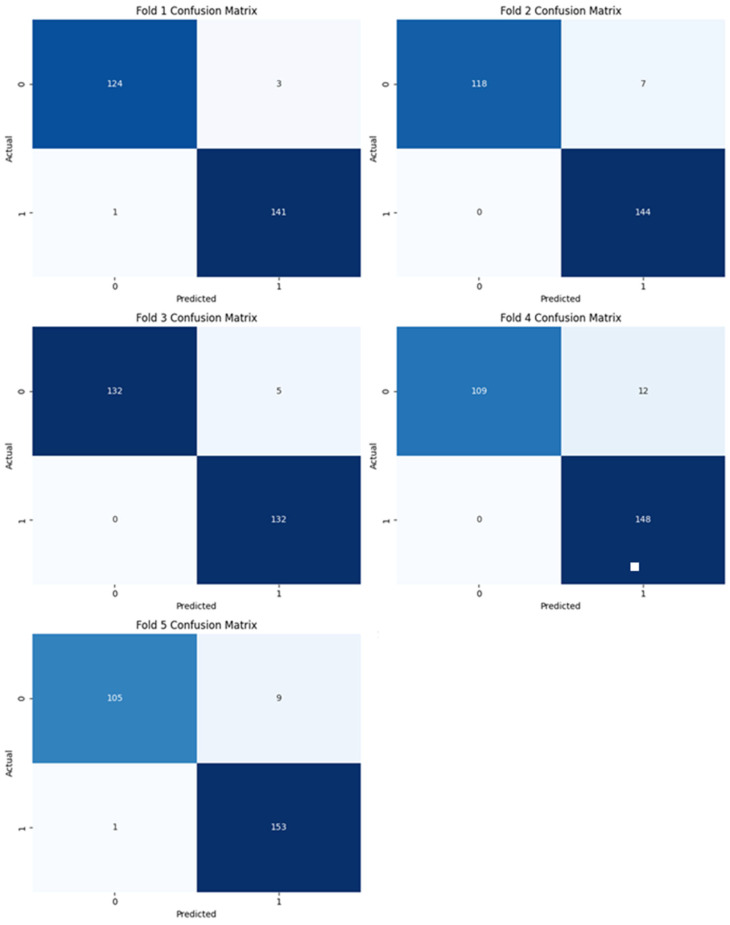
Confusion matrix of Model_1.

**Figure 11:**
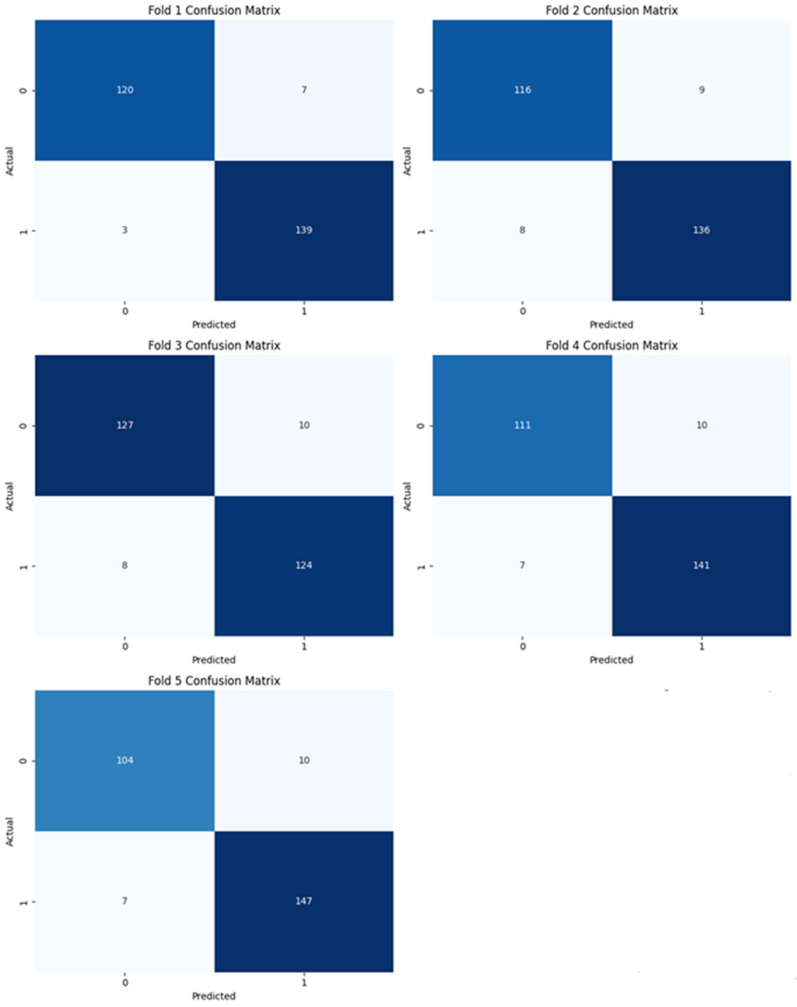
Confusion matrix of Model_2.

In this section, we have presented the performance evaluation of Model_1 and Model_2 based on various metrics. The results are detailed in Table 2, which outlines key performance indicators for both models. Further insights into their performance are visualized in Figure 6, which illustrates the performance metrics of Model_1 and Model_2. Additionally, the AUC-ROC curve for fold_1 of each model is shown in Figure 7, providing a comparative view of their discriminative capabilities. The loss function variations across five folds for both models are depicted in Figure 8 and Figure 9, highlighting the stability and convergence of each model during training. To evaluate the models’ classification accuracy, the confusion matrices for Model_1 and Model_2 are presented in Figure 10 and Figure 11, respectively. Finally, Table 3 compares the results of the proposed models with various state-of-the-art methods, offering a broader context for their performance.

Figure 6 presents a comparative analysis of performance metrics for two classification models—Model_1 (Support Vector Machine) and Model_2 (Autoencoder + Fully Connected Neural Network)—evaluated using 5-fold cross-validation. The metrics visualized include Accuracy, Precision, F1 Score, Specificity, Recall, and Area Under the ROC Curve (AUC) and PRC curve and area under the curve.

Figure 7 illustrates the Receiver Operating Characteristic (ROC) curves for both classification models evaluated in this study: Model_1 (Support Vector Machine-SVM) and Model_2 (Deep Learning model using Autoencoder + FCNN). The ROC curve is a graphical representation of the trade-off between the true positive rate (sensitivity) and false positive rate (1-specificity) across various threshold settings. The left plot shows the ROC curve for the SVM model, which exhibits strong classification performance with an AUC (Area Under the Curve) of 0.99. The curve closely hugs the top-left corner, indicating a high true positive rate with a low false positive rate. The right plot displays the ROC curve for the deep learning model, which also achieves an AUC of 0.99, matching the SVM in overall discriminative ability. However, the DL model shows an even sharper rise near the *y*-axis and maintains a true positive rate close to 1.0 for the majority of the curve, suggesting potentially superior early sensitivity at lower false positive rates.

The “loss curves” in Figure 8 are not actual train/validation losses. SVMs are not trained in epochs like neural networks. There is no iterative training over batches with intermediate loss values like in deep learning. Instead, we are plotting the sorted decision scores from the SVM output for test samples in each fold, which is more of a margin confidence visualization rather than a learning curve. A decision score plot visualizes the raw output of the SVM decision function, which represents the distance of each sample from the separating hyperplane, that is, the margin.

Figure 9 shows the training and validation loss curves for the deep learning model (Autoencoder + FCNN) over five-fold cross-validation. Each subplot corresponds to a specific fold (Fold 1 to Fold 5), displaying how the model’s loss evolved across epochs during training. The loss curves demonstrate that the Autoencoder + FCNN model was trained efficiently and generalizes well across all folds, reinforcing the performance gains observed in the previous metrics.

Figure 10 illustrates the confusion matrices for each of the five folds used in cross-validation for Model_1. Each matrix provides a detailed view of the model’s classification performance by showing the number of true positives (TP), true negatives (TN), false positives (FP), and false negatives (FN) for binary classification.

Figure 11 illustrates the confusion matrices for each of the five folds used in cross-validation for Model_2. Each matrix provides a detailed view of the model’s classification performance by showing the number of true positives (TP), true negatives (TN), false positives (FP), and false negatives (FN) for binary classification.

## 5. Discussion

This study proposed a deep learning-based framework for the classification of schizophrenia using EEG signals. The approach combined Markov Transition Field (MTF) transformation with deep feature extraction using VGG-16, followed by dimensionality reduction through an autoencoder and final classification using a neural network (NN). A Support Vector Machine (SVM) classifier was also used for comparison. The experimental results demonstrate that the autoencoder + NN model achieved the best performance, with an accuracy of 98.51%, precision of 97.92%, recall of 100.00%, F1-score of 98.60%, and AUC of 99.92%. The SVM classifier also produced comparable results, with an accuracy of 96.28% and AUC of 99.62%. Table 2 demonstrates the comparison of the proposed model with the various state-of-the-art models, thus noting the important contribution provided by the proposed model in the field of diagnosis of schizophrenia using AI models.

### 5.1. Comparative Analysis of SVM and Neural Network Model

In the context of EEG data analysis, Support Vector Machines (SVMs) are particularly advantageous in scenarios where the dataset is relatively small to moderately sized, which is often the case in clinical studies or experiments with limited participants. SVMs perform well when the feature space is well-structured and can be made linearly separable through appropriate transformations, such as kernel functions. Additionally, their relatively simple structure and mathematical formulation make them highly interpretable and computationally efficient, which is beneficial for applications requiring real-time decision-making or deployment on resource-constrained devices. In comparison, Neural Networks (NNs)—especially deep architectures—are more suitable when working with large-scale or high-dimensional EEG datasets that contain rich spatial and temporal patterns. These models excel in capturing non-linear relationships within the data and are capable of learning complex features directly from raw or minimally processed signals. Neural networks are ideal for end-to-end learning tasks where manual feature engineering is either infeasible or suboptimal.

### 5.2. Model Explainability Using SHAP

SHAP [18] stands for SHapley Additive exPlanations, a method in Explainable AI (XAI) that uses game theory to explain the output of machine learning models. It assigns each input feature a value representing its contribution to the model’s prediction, helping to understand which features are most influential. SHAP values are based on Shapley values, a concept from game theory that measures each player’s contribution to the outcome. 

**Figure 12:**
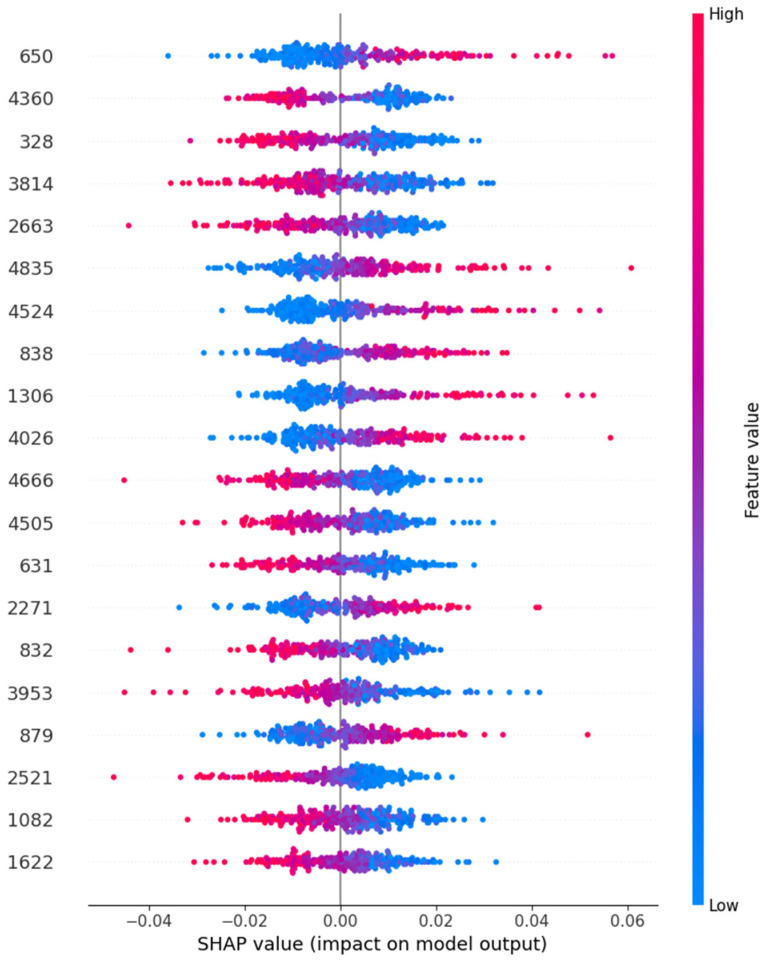
Model’s explainability using SHAP.

The VGG-16 model outputs a range of feature vectors, which were then evaluated using SHAP. To better understand the decision-making process of the Autoencoder + Neural Network (NN) model, we utilized SHAP (SHapley Additive exPlanations) values. SHAP values offer insights into how individual features contribute to the overall model prediction. Figure 12 visualizes the SHAP values for various features used in the model.

SHAP values for the Autoencoder + Neural Network model, showing the impact of individual features on the model’s output. The colors represent the feature value, where blue corresponds to low values and pink to high values. Positive and negative SHAP values indicate the direction of influence on the model’s prediction.

The SHAP plot demonstrates that certain features significantly impact the model’s output, with some features having a higher positive or negative impact. By interpreting the SHAP values, we gain insights into the most important features for classifying schizophrenia, which can guide future model refinement and feature selection.

The plot presents the SHAP summary plot, which provides insight into the contribution of individual features to the model’s predictions. Features such as **650, 4360, and 328** exhibit the highest SHAP values, indicating they have the greatest impact on the model’s decision-making process.

The **feature 650** consistently shows a wide distribution of SHAP values, with both high and low values influencing the model output in opposite directions. This suggests it may act as a key discriminator between different classes. The red (high feature value) points being spread towards positive SHAP values imply that higher values of this feature are associated with a higher likelihood of a positive classification (e.g., presence of a condition), whereas blue (low feature value) points shift predictions toward the negative class.

Likewise, **feature 4360** demonstrates a strong effect where higher feature values negatively impact the prediction, suggesting that this particular signal characteristic could be indicative of a non-target class (e.g., normal state).

In contrast, some features like **feature 838 or 1082** appear further down in the plot and show tighter SHAP distributions around zero, indicating that these have a relatively lower influence on the model’s decision-making. These insights underline the model’s ability to detect subtle EEG variations across specific channels or temporal segments.

### 5.3. Advantages of the Proposed Model

- High Accuracy and Robust Performance: The model achieved exceptionally high classification performance (98.51% accuracy, 100% recall), indicating strong potential for reliable schizophrenia detection.
- Effective Feature Representation: By transforming EEG signals into Markov Transition Fields (MTFs) and extracting deep features through VGG-16, the model captures complex spatial–temporal patterns associated with brain activity.
- Dimensionality Reduction: The use of an autoencoder significantly reduces feature dimensionality while retaining critical information, leading to a more efficient and noise-resilient model.
- Model Explainability: SHAP (SHapley Additive exPlanations) analysis adds a layer of explainability, making it easier to understand which features contribute most to the classification outcome—a crucial step toward clinical adoption.
- Comparative Versatility: The framework is flexible, as demonstrated by the competitive performance of both deep learning (Autoencoder + NN) and classical (SVM) classifiers.

### 5.4. Limitations of the Proposed Model

- Binary Classification Scope: The model currently addresses only binary classification (schizophrenia vs. healthy), which limits its applicability in diagnosing other psychiatric or neurological disorders.
- Dataset Constraints: Results are based on a single dataset with a limited number of subjects. The model’s performance and generalizability across diverse populations or recording settings remain to be tested.
- Computational Requirements: Deep learning models, particularly those involving VGG-16 and autoencoders, require substantial computational resources and may not be feasible in all clinical environments.
- Black-Box Nature: Despite SHAP-based insights, deep learning models are still relatively opaque compared to traditional statistical methods, which may hinder clinician trust and adoption.

### 5.5. Practical Applications

- Clinical Decision Support: The proposed system can serve as a valuable tool to assist psychiatrists in early and objective diagnosis of schizophrenia, reducing reliance on subjective assessments.
- Screening in Remote or Resource-Limited Settings: With appropriate optimization, this model could enable preliminary screening using portable EEG devices in underserved regions.
- Neuropsychiatric Research: The framework can aid in studying neural biomarkers of schizophrenia, helping identify key EEG patterns linked to the disorder.

### 5.6. Challenges in Real-World Deployment

- Standardization of EEG Acquisition: EEG signals are sensitive to noise, electrode placement, and individual variability. Standardizing data collection protocols is essential for reliable results.
- Regulatory and Ethical Concerns: Deployment in clinical settings would require regulatory approval and careful handling of patient data to ensure privacy and ethical use.
- Model Adaptability: Models need to adapt to individual variability, comorbid conditions, and medication effects, which can alter EEG patterns.
- Clinician Acceptance: To be integrated into routine practice, the model must be transparent, easy to use, and complement (not replace) clinical expertise.

## 6. Conclusions and Future Work

This study presents an effective deep learning framework for schizophrenia classification using EEG signals, utilizing the strength of Markov Transition Field (MTF) transformation and deep feature extraction through VGG-16. The extracted features were further reduced using an autoencoder and classified using a neural network (NN). The Support Vector Machine (SVM) algorithm also showed comparable results. Among these, the autoencoder + NN pipeline demonstrated superior performance, giving an accuracy of 98.51%, a precision of 97.92%, a recall of 100.00%, an F1-score of 98.60%, and an AUC of 99.92%. The SVM classifier also yielded promising results with an accuracy of 96.28% and an AUC of 99.62%.

To provide interpretability of the deep model, SHAP (SHapley Additive exPlanations) analysis was employed, revealing the most influential features impacting classification decisions. This analysis enhances transparency and aids in understanding the relationship between EEG-derived patterns and schizophrenia-related neural activity.

In future work, this framework could be expanded to multi-class classification involving different psychiatric disorders, enabling broader diagnostic applicability. The development of a multi-modal dataset comprising EEG data with speech or video data can help in utilizing the natural language processing area of AI. With the new dataset, the development and testing of new models or newer versions of existing models is an area of exploration and research.

## Figures and Tables

**Table 1:** Confusion matrix representation.

	Predicted Positive	Predicted Negative
Actual Positive	True Positive (TP)	False Negative (FN)
Actual Negative	False Positive (FP)	True Negative (TN)

**Table 2:** Performance metrics of Model_1 and Model_2.

Performance Metrics		Accuracy	Precision	F1 Score	Specificity	Recall	AUC	PRC AUC
Model_1 (SVM)	Fold-1 Fold-2 Fold-3 Fold-4 Fold-5	**96.28** 93.68 93.31 93.68 93.66	**95.21** 93.79 92.54 93.38 93.63	**96.53** 94.12 93.23 94.31 94.53	**94.49** 92.80 92.70 91.74 91.23	**97.89** 94.44 93.94 95.27 95.45	**99.62** 98.57 98.16 98.84 98.49	**96.52** 94.00 93.21 94.26 94.50
Model_2 (Autoencoder + FCNN)	Fold-1 Fold-2 Fold-3 Fold-4 Fold-5	**98.51** 97.40 98.14 95.54 96.27	**97.92** 95.36 96.35 92.50 94.44	**98.60** 97.63 98.14 96.10 96.84	**97.64** 94.40 96.35 90.08 92.11	99.30 **100.00** **100.00** **100.00** 99.35	**99.92** 99.69 99.91 99.78 99.74	**98.60** 97.65 98.14 96.09 96.83

**Table 3:** Comparison of results of the proposed model with the various state-of-the-art models.

Authors	Dataset	Classification Algorithm	Performance Metrics (%)
Aslan et al., 2020 [10]	Open-access Schizophrenia EEG database provided by MV Lomonosov Moscow State University and the Institute of Psychiatry and Neurology, Warsaw, Poland	Deep features were extracted from STFT images using a pre-trained VGG-16 convolutional neural network model	Accuracy (Dataset1) = 95.00 Accuracy (Dataset 2) = 97.00
**Proposed model**	Open-access Schizophrenia EEG database provided by MV Lomonosov Moscow State University	The approach combined Markov Transition Field (MTF) transformation with deep feature extraction using VGG-16, followed by dimensionality reduction through an autoencoder and final classification using a neural network (NN) (Model_2). A Support Vector Machine (SVM) (Model_1) classifier was also used for comparison.	Model_1: Accuracy = 96.28, precision = 95.21, Specificity = 94.49 recall = 97.89, F1-score = 96.53, and AUC = 99.62 Model_2: Accuracy = 98.51, precision = 97.92, Specificity = 97.64 recall = 100.00, F1-score = 98.60, and AUC = 99.92

## Data Availability

This study uses an open-source Schizophrenia EEG database provided by MV Lomonosov Moscow State University. [EEG Database—Schizophrenia] [http://brain.bio.msu.ru/eeg_schizophrenia.htm] (accessed on 20 June 2025).
